# Patients With Epilepsy Who Underwent Epilepsy Surgery During the COVID-19 Pandemic Showed Less Depressive Tendencies

**DOI:** 10.3389/fneur.2021.677828

**Published:** 2021-05-04

**Authors:** Keiko Niimi, Ayataka Fujimoto, Keishiro Sato, Hideo Enoki, Tohru Okanishi

**Affiliations:** Comprehensive Epilepsy Center, Seirei Hamamatsu General Hospital, Hamamatsu, Japan

**Keywords:** Zung self-rating depression scale, pandemic, epilepsy surgery, COVID-19, SARS-CoV-2

## Abstract

**Introduction:** Our hypothesis in this study was that differences might exist between patients with epilepsy (PWE) who underwent epilepsy surgery before and within the period of the coronavirus disease 2019 (COVID-19) pandemic. The purpose of this study was to compare results of the Zung Self-Rating Depression Scale (SDS) between PWE who underwent epilepsy surgery before and during the pandemic period.

**Methods:** Participants were PWE who underwent open cranial epilepsy surgery between February 2019 and February 2021 in our hospital. Patients who underwent surgery in the first half of this period, between February 2019 and January 2020, were defined as the pre-pandemic period group (pre-Group) and those treated in the second half, between February 2020 and February 2021, were categorized as the pandemic period group (within-Group). All patients completed the SDS before surgery, and scores were compared between groups.

**Results:** SDS score was significantly higher in the pre-Group than in the within-Group (*p* = 0.037). Other factors, including age (*p* = 0.51), sex (*p* = 0.558), epilepsy duration from onset to SDS score evaluation (*p* = 0.190), seizure frequency (*p* = 0.794), number of anti-seizure medications (*p* = 0.787), and intelligence quotient (*p* = 0.871) did not differ significantly between groups.

**Conclusion:** SDS score was higher in the pre-pandemic group than in the within-pandemic group, which may indicate that PWE with less-positive outlooks may be less likely to seek medical attention during stressful periods.

## Highlights

- PWE who underwent surgery during the pandemic period showed less depressive symptoms.
- PWE with depressive tendencies might be at risk during stressful periods.
- Technology might offer hope for PWE showing depressive tendencies.

## Introduction

The coronavirus disease 2019 (COVID-19) pandemic caused by severe acute respiratory syndrome coronavirus 2 (SARS-CoV-2) started in 2019, and has shown three peaks in Japan since the beginning of 2020.

Previously, as pandemics in the 20th century, we experienced the Spanish flu, acquired immunodeficiency syndrome (AIDS) and highly pathogenic avian influenza viruses (1–4). However, in the 21st century, the frequency of epidemics seems to have increased markedly (5–9). Regardless of the cause, the possibility of pandemics occurring more frequently in the near future must naturally be considered.

Patients with epilepsy (PWE) are known to have been suffering during the calamity of COVID-19 due to anxiety and depression (10). In particular, PWE with surgically remediable epilepsy are known to be vulnerable to psychiatric disturbance before and after surgery, even when societal calamities are not present. Since the adequate treatment of psychiatric comorbidities increases the likelihood of seizure freedom and optimizes the psychosocial benefits afforded by epilepsy surgery (11), provision of psychological care to PWE must be a priority. At the same time, continuity of treatment for PWE is also necessary. The idea of continuing business functions in society has already been described in 2012 by the International Organization for Standardization (ISO) as ISO22301 (https://www.iso.org/standard/75106.html). This ISO standard describes how to continually manage business in an organization to protect against the occurrence of disruptive incidents. These underlying principles are also applicable to healthcare businesses and our hospital follows this standard.

We have already described a retrospective study showing the importance of local interdisciplinary care for PWE (12). We have also shown that SARS-CoV-2 did not influence the volume of epilepsy surgeries in our facility (13). From these efforts, approaches to the calamity from the healthcare perspective might improve such negative situations for PWE. However, in the real world, worsening of seizure frequency and postponement of medical examinations are commonplace experiences for many PWE during the COVID-19 pandemic (14). If many PWE postponed adequate medical treatments during the pandemic, psychological differences might exist between PWE who did not postpone treatments and underwent epilepsy surgery during the pandemic and PWE who underwent epilepsy surgery before the pandemic.

Our hypothesis for the present study was thus that psychological differences might exist between PWE who underwent epilepsy surgery before and during the COVID-19 pandemic. The purpose of this study was to compare results from a depression scale among PWE who underwent epilepsy surgery before and during the COVID-19 pandemic.

## Methods

### Study Design and Ethics Approval

The ethics committee at Seirei Hamamatsu General Hospital, Japan, approved the protocol for this retrospective study (approval no. 3578), which was performed in accordance with the principles of the Declaration of Helsinki. Subjects in this study were identified from a review of the electronic medical records for patients who had undergone epilepsy surgery between February 2019 and February 2021 in the Comprehensive Epilepsy Center at Seirei Hamamatsu General Hospital.

### Clinical Information

We collected information from patients who underwent epilepsy surgery between February 2019 and February 2021, as the first half of this period between February 2019 and January 2020 was pre-pandemic and the second half between February 2020 and February 2021 was just within the pandemic period in Japan. Patient age was recorded at the time of depression scale evaluation.

Data were obtained from all 32 patients who underwent open cranial epilepsy surgery for medically intractable epilepsy between February 2019 and February 2021 in our hospital. Among this population, inclusion criteria were: (1) age ≥18 years at evaluation, as the depression scale used was the Zung Self-Rating Depression Scale [SDS], which is adapted for individuals ≥18 years old (15); and (2) full intelligence quotient (IQ) ≥60. Among this population, exclusion criteria were: (1) patients who had undergone vagus nerve stimulation (VNS) therapy; and (2) patients who exhibited psychogenic non-epileptic seizures (PNES) (16) or other non-stereotypical activities.

As intellectual disorder and depression are sometime difficult to differentiate, such as in depressive cognitive disorders (17), patients with more than moderate intellectual disorder were allowed to enroll in this study. As VNS therapy has antidepressant effects (18), patients with VNS devices were excluded. All patients underwent long-term video electroencephalography and stereotypical epileptic seizures were captured. Based on the stereotypical seizure semiology, PWE who had experienced non-stereotypical activities were excluded because these non-stereotypical activities were regarded as possible PNES or non-epileptic events.

### Depression Scale as the Primary Outcome Measurement

The SDS test is a 20-item self-reported questionnaire in common use as a screening tool, covering affective, psychological, and somatic symptoms associated with depression. The questionnaire takes 5–10 min to complete, and items are framed in terms of positive and negative statements. The total score is derived as the sum of scores for the individual item scores, ranging from 20 to 80. Patients who undergo epilepsy surgery in our institution undergo SDS before the surgery. We divided the enrolled patients into two groups: those who underwent surgery in the first half of the period, categorized as during the pre-pandemic period group between February 2019 and January 2020 (pre-Group); and those who underwent surgery in the second half of the period, categorized as during the pandemic period between February 2020 and February 2021 (within-Group). We compared SDS scores between these two groups.

### Seizure Frequency, Duration From Epileptic Seizure Onset to SDS Score Evaluation, and Anti-seizure Medications as Secondary Outcome Measurements

We also compared seizure frequency, duration from onset to SDS score evaluation, and number of anti-seizure medications (ASMs) between the pre-Group and within-Group. Seizure frequency was classified as: (1) daily; (2) weekly; (3) monthly; or (4) yearly. These secondary outcomes were chosen as factors potentially related to anxiety or depressive disorders (19).

### Statistical Analyses

The Mann-Whitney *U*-test and Student's *t*-test were used in this study, as appropriate. Statistical significance was set at the level of *p* < 0.05. Analyses were conducted using Sigma plot (Systat Software, San Jose, CA, USA).

## Results

Clinical information and results of the SDS are shown in Table 1.

**Table 1:** Clinical information and SDS scores.

	**Pre-group (*n* = 10)**	**Within-group (*n* = 9)**	***p*-value**
Sex (female), *n*	3 (30%)	4 (44%)	0.558
Age at evaluation (years)	mean 30.7, SD 14.2, median 27	mean 34.7, SD 11.5, median 37	0.51
Type of epilepsy	7 TLE, 2 FLE, 1 OLE	7 TLE, 2 FLE	na
IQ	mean 85.8, SD 20.7, median 89.5	mean 84.3, SD 18.1, median 86	0.871
SDS score	mean 39.9, SD 6.4, median 41.5	mean 33.2, SD 6.45, median 32	0.037^*^
Seizure frequency	mean 2.6, SD 0.84, median 3.0	mean 2.67, SD 1.12, median 3	0.794
Duration from onset to SDS evaluation (m)	mean 115.5, SD 113.2, median 72	mean 193.3, SD 155.7, median 144	0.19
No. of ASMs	mean 2, SD 0.94, median 2	mean 1.89, SD 0.928, median 2	0.787

*SD, standard deviation; SDS, self-rating depression scale; TLE, temporal lobe epilepsy; FLE, frontal lobe epilepsy; OLE, occipital lobe epilepsy; IQ, intelligence quotient; ASM, anti-seizure medication; m, months. Seizure frequency was classified as: (1) daily; (2) weekly; (3) monthly; or (4) yearly*.

* *significant difference, p < 0.05*.

Seven female PWE and 12 male PWE (mean age at evaluation, 32.6 years; median age, 35.0 years; range, 18–59 years; standard deviation, 12.8 years; confidence interval of the mean, 6.16 years) fulfilled the inclusion criteria. None of the patients exhibited PNES. Table 2 shows the ASMs used by the patients.

**Table 2:** Use of ASMs.

	**Pre-group**							**Within-group**				
Anti seizure medications	LCM	LEV	PER	CBZ	CLB	TPM	LTG	LCM	LEV	PER	CBZ	CLB
No. of PWE	7	5	4	1	1	1	1	6	5	4	1	1

*LCM, Lacosamide; LEV, Levetiracetam; PER, Perampanel; CBZ, Carbamazepine; CLB, Clobazam; TPM, Topiramate; LTG, Lamotrigine*.

### Primary Outcome Measurement

SDS scale score was significantly higher in the pre-Group than in the within-Group (*p* = 0.037). No other factors, including age (*p* = 0.51), sex (*p* = 0.558), or IQ (*p* = 0.871), showed significant differences between groups (Table 1).

### Secondary Outcome Measurements

Epilepsy duration from onset to SDS score evaluation (*p* = 0.190), seizure frequency (*p* = 0.794), and number of ASMs (*p* = 0.787) all showed no significance differences between groups (Table 1).

## Discussion

SDS scale score was significantly higher in the pre-Group than in the within-Group. Even though scores were not within the diagnostic range for depression, the pre-Group was relatively closer to the range for depressive symptoms than the within-Group. This might be because PWE with a more positive outlook were less likely to put off epilepsy surgery even during the pandemic period.

Rayner and Wilson (11) reported that a less compromised psychiatric profile may contribute to better outcomes of epilepsy surgery. Even though we did not provide special treatments for psychiatric comorbidities for enrolled PWE, the fact that PWE with a less negative outlook underwent epilepsy surgery during the pandemic in this study might partially support this theory by Rayner and Wilson. Given the disaster-preparedness measures taken by our facility, the continuity plan might have worked. As Japan experiences relatively frequent disasters, such as earthquakes, typhoons, and floods, many facilities in Japan might prepare for such disruptive events. The other effort was that as our local interdisciplinary system for epilepsy treatment became established (13), the flow cycle for PWE among local general physicians and our epilepsy center was able to be maintained. Based on these approaches to the calamity that might be decreasing psychological stresses among surgical candidates, thereby improving such negative situations for PWE, epileptologists might not hesitate to perform epilepsy surgery even during a pandemic.

Conversely, about 30% of PWE are considered to have depressive symptoms (20). Many of these patients might be more strongly influenced by psychological factors than by the epilepsy itself (21). From our results and those of previous reports, the possibility should be considered that some PWE might neglect beneficial advanced medical care such as epilepsy surgery under disruptive situations.

As interventions in medical treatments, including for epilepsy, have a certain order of priority (22), some PWE might be classified as having non-urgent disorders (23), despite they might have severe conditions such as COVID-19-related status epilepticus (24). However, using advanced technologies such as telemetry (10, 25), home-video recordings (26) and tele-neuropsychology tests (27) should be implemented to maximize efficient provision of appropriate medical interventions.

A key limitation of this study was the small number of PWE in each group. However, the suggestion that some PWE might be neglected under various circumstances is important and worth exploring further in future work.

SDS scores obtained from multiple centers before and during the pandemic and other societal circumstances should be analyzed in future investigations.

## Conclusion

SDS score was higher in the pre-pandemic group than in the within-pandemic group, which may indicate that PWE with less-positive outlooks may be less likely to seek medical attention during periods of societal or personal stress.

## Data Availability Statement

The original contributions generated for this study are included in the article/supplementary material, further inquiries can be directed to the corresponding author/s.

## Ethics Statement

The studies involving human participants were reviewed and approved by The ethics committee of Seirei Hamamatsu General Hospital. The patients/participants provided their written informed consent to participate in this study.

## Author Contributions

KN, AF, HE, KS, and TO: acquisition of data. AF, TO, and HE: analysis and interpretation of data. AF: epilepsy surgery. All authors contributed to the article and approved the submitted version.

## Conflict of Interest

The authors declare that the research was conducted in the absence of any commercial or financial relationships that could be construed as a potential conflict of interest.
